# Current Cervical Cancer Status in Resource-Limited Somalia: a Systematic Review of Burden, Screening Findings, and Knowledge Gaps

**DOI:** 10.1007/s44197-026-00526-x

**Published:** 2026-02-26

**Authors:** Najib Isse Dirie, Mulki Mukhtar Hassan, Mohamed Mustaf Ahmed, Jihaan Hassan, Bashiru Garba, Saleh Abdulkadir Saeed Al-Duais, Daniela Massi

**Affiliations:** 1 Department of Urology, Faculty of Medicine and Health Sciences, Dr. Sumait Hospital, SIMAD University, Mogadishu, Somalia; 2 SIMAD Institute for Global Health (SIGHt), SIMAD University, Mogadishu, Somalia; 3 Faculty of Medicine and Health Sciences, SIMAD University, Mogadishu, Somalia; 4 Department of Pediatrics, Faculty of Medicine and Health Sciences, Dr. Sumait Hospital, SIMAD University, Mogadishu, Somalia; 5 Department of Public Health, Faculty of Medicine and Health Sciences, SIMAD University, Mogadishu, Somalia; 6 Section of Pathology, Department of Health Sciences, University of Florence, Florence, Italy

**Keywords:** Cervical cancer, Human papillomavirus, Somalia, Screening, Healthcare professionals, Women's health

## Abstract

**Introduction:**

Cervical cancer is the second most frequent cancer among Somali women, with approximately 1,055 diagnoses and 812 deaths annually; however, evidence to guide control efforts is scarce. This systematic review synthesizes the burden, screening findings, and knowledge gaps to inform policy and practice in Somalia’s resource-limited settings.

**Methods:**

Following PRISMA 2020, we searched PubMed, Scopus, Embase, ScienceDirect, DOAJ, Cochrane Library, and Google Scholar without date limits for English, peer-reviewed studies from Somalia reporting prevalence, incidence, knowledge, screening, and prevention. Two reviewers independently extracted the data and performed the JBI critical appraisal for cross-sectional studies. Given the heterogeneity, we conducted a narrative synthesis.

**Results:**

Seven cross-sectional studies (2019–2024) with 3,797 participants from Mogadishu, Hargeisa, and Bosaso were included in this review. Across facility-based studies, cervical cancer accounted for 6.7% of all cancers and 13.3% of female cancers, while screening positivity among women attending screening services ranged up to 15.7%. The prevalence of cervical cancer ranged from 6.7% to 15.7%. Among 189 women sampled in Mogadishu (clinic-based), 31.7% were HPV DNA-positive, including 19.6% high-risk types (HPV16 13.8%, HPV18 5.3%); these findings may not represent national HPV prevalence. Among healthcare professionals, 73% had good theoretical knowledge; however, only 24% had treated a cervical-cancer patient, 22% reported receiving specific training, and only 2% of female providers had undergone Pap testing. Women’s awareness varied widely (43.7–97.7%), and misconceptions were common. In one facility-based screening study, VIA positivity was 7.6% compared with 5.1% for Pap smear (*p* = 0.004). Among women who were VIA-positive and underwent confirmatory assessment, 73.9% had CIN on colposcopy/biopsy; however, biopsy-confirmed CIN2+/cancer outcomes by each test and verification pathways were not fully reported, limiting direct comparison of diagnostic performance.

**Conclusions:**

Somalia faces a high burden of cervical cancer and HPV infection, compounded by limited screening coverage, training gaps among providers, and misinformation among women about HPV vaccination. Priority actions include establishing a national cancer registry, expanding HPV vaccination, scaling context-appropriate screening (VIA and HPV testing), delivering targeted public education, and strengthening provider training especially for female frontline staff using culturally sensitive approaches to improve uptake and outcomes.

**Supplementary Information:**

The online version contains supplementary material available at 10.1007/s44197-026-00526-x.

## Introduction

Cervical cancer is a major global health challenge, ranking as the fourth most common cancer among women worldwide, with approximately 660,000 new cases and 350,000 deaths in 2022 [1–3]. The burden of disease is disproportionately concentrated in low- and middle-income countries, driven by limited access to HPV vaccination, inadequate screening services, and broader socioeconomic determinants [1, 4, 5]. Persistent infection with human papillomavirus (HPV), particularly types 16 and 18, is the primary cause of cervical cancer [6–8] and women living with HIV face a substantially increased risk [1]. In sub-Saharan Africa, cervical cancer remains the most common malignancy among women in many countries and is associated with poor outcomes, with a reported 5-year survival rate of approximately 35%, reflecting late-stage diagnosis, weak health systems, and structural barriers to care [6, 9, 10]. The region reports alarmingly low survival rates, with a meta-analysis revealing a 5-year survival rate of merely 35%, which is significantly lower than the global average [6]. This poor prognosis stems from multiple factors, including late-stage presentation, limited healthcare infrastructure, geographical barriers to care, and high HIV prevalence. Eastern Africa has a mortality rate of 35 per 100,000 women, highlighting the severity of the crisis in this region [6].

Somalia faces particularly severe challenges in cervical cancer prevention and control. Recent estimates indicate that 1,055 Somali women are diagnosed annually, with 812 deaths, making cervical cancer the second most frequent cancer among women aged 15–44 years [11]. The age-standardized incidence rate is 25.1 per 100,000 women, with a mortality rate of 20.2 per 100,000, underscoring the magnitude of the problem [11]. Despite this burden, screening coverage remains extremely limited, with only 1–2% of eligible women ever screened [11]. Importantly, no population-based cervical cancer prevalence studies have been conducted in Somalia; available evidence derives from facility-based cancer registries and screening attendees and should not be interpreted as national prevalence estimates. Data on HPV prevalence and genotype distribution in the general population are also scarce [12]. Although healthcare professionals demonstrate relatively good theoretical knowledge of cervical cancer, gaps remain in vaccine awareness and service availability, with most providers reporting that HPV vaccination is inaccessible to their patients [13].

Given Somalia’s fragile healthcare system, shaped by decades of conflict and instability [14]. synthesizing existing evidence on cervical cancer burden, screening findings, and knowledge gaps is essential. Such understanding is critical to informing policy development, guiding resource allocation, and designing culturally appropriate and context-specific interventions. Therefore, this systematic review aims to comprehensively map the available evidence on cervical cancer in Somalia, focusing on disease burden, screening practices, and knowledge gaps, to support efforts to strengthen cervical cancer control in this resource-limited setting.

## Methods

### Search Strategy and Information Sources

A comprehensive systematic review was conducted following the 2020 Preferred Reporting Items for Systematic Reviews and Meta-Analyses (PRISMA) guidelines [15]. The PRISMA 2020 checklist was used to ensure transparency, completeness, and methodological rigor throughout the review process [S1]. Multiple electronic databases were systematically searched to identify relevant studies on cervical cancer in Somalia, including PubMed, Scopus, Google Scholar, Embase, ScienceDirect, the Directory of Open Access Journals (DOAJ), and the Cochrane Library. The search was conducted without date restrictions to capture all the available evidence. A comprehensive search strategy was implemented using keywords ‘cervical cancer,’ ‘human papillomavirus or HPV,’ and ‘Somalia or Somali,’ among others, in combinations. The search spanned from inception to February 2025, with no date limits. Database-specific search strategies were also developed using controlled vocabularies and free-text terms. For PubMed, the search string incorporated MeSH terms: (“cervical cancer” OR “human papillomavirus” OR HPV [MeSH Terms]) AND (Somalia OR Somaliland OR Mogadishu OR Hargeisa [MeSH Terms]). In Scopus, the title, abstract, and keyword fields were searched using the following terms: (TITLE-ABS-KEY (“cervical cancer” OR “human papillomavirus” OR HPV) AND TITLE-ABS-KEY (Somalia OR Somaliland OR Mogadishu OR Hargeisa)). Similar search strategies were adapted for other databases, combining cervical cancer-related terms with Somalia-specific geographic terms to identify relevant studies.

### Eligibility Criteria

Studies were selected based on predefined inclusion and exclusion criteria. For inclusion, we added peer-reviewed original research articles (e.g., cross-sectional, cohort and case-controlled studies) that presented new data; we excluded case reports, conference abstracts, commentaries, and reviews. Only peer-reviewed publications in English were considered because no relevant Somali-language publications were identified and translation resources were limited. The review excluded non-peer-reviewed articles, reviews, commentaries, letters, perspectives, editorials, conference abstracts, and gray literature, including unpublished theses and reports.

### Study Selection Process

**Fig. 1:**
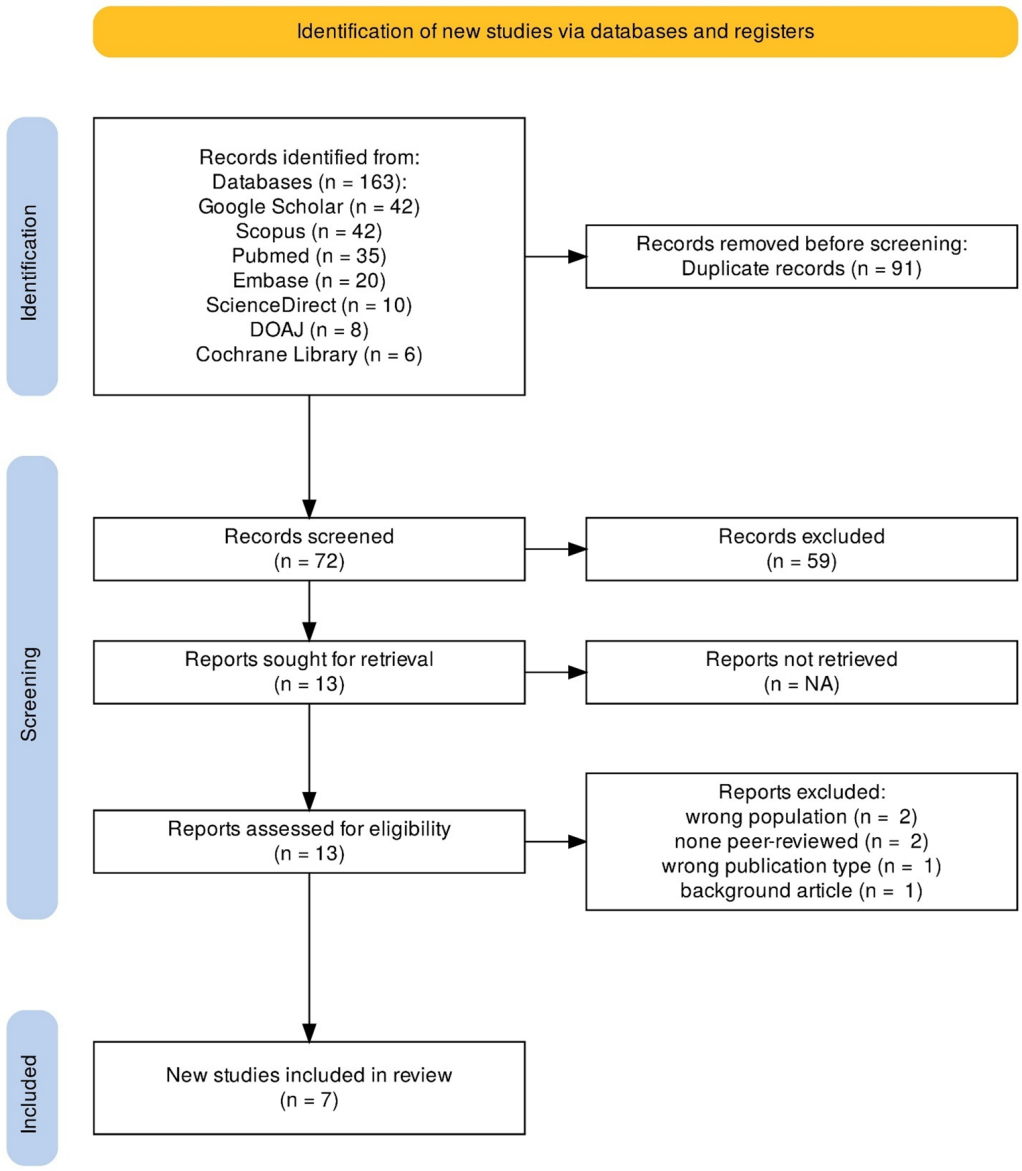
PRISMA flow diagram for the systematic review on cervical cancer in Somalia

Two reviewers (MMA and MMH) independently screened titles and abstracts, followed by full-text assessment for eligibility. Any disagreements were resolved through discussion and consensus, with consultation of a third reviewer (NID) when necessary. The study selection process was systematic and involved multiple stages (Fig. 1). Initially, 163 records were identified across all databases, with Google Scholar and Scopus each yielding 42 records, PubMed yielding 35 records, Embase 20 records, ScienceDirect 10 records, DOAJ 8 records, and Cochrane Library 6 records. After identification, 91 duplicate records were removed. The remaining 72 records underwent title and abstract screening against the eligibility criteria. This screening process excluded 59 records that did not meet the inclusion criteria. Subsequently, 13 reports were retrieved in full text and assessed for eligibility. During this assessment, six reports were excluded for specific reasons: two studies involved the wrong population, two were not peer-reviewed, one was the wrong publication type, and one was excluded due to having a different background. Ultimately, 7 studies met all eligibility criteria and were included in the final review.

### Data Extraction and Synthesis

A standardized data extraction form was developed to systematically collect relevant information from each included study. Two independent reviewers (MMA and MMH) extracted data to ensure accuracy and completeness. The extracted data included author(s), year, study objective, study design, sample size, population/setting, region, key findings, screening methods and recommendations. Given the heterogeneity of study methods, populations, and outcomes, quantitative meta-analysis was not appropriate; instead, we conducted a narrative synthesis, as recommended for systematically reviewing diverse evidence [16]. Given the heterogeneity of the included studies in terms of design, population, and outcomes, a narrative synthesis approach was employed rather than a meta-analysis. The synthesis focused on identifying patterns, similarities, and differences across studies to provide a comprehensive understanding of cervical cancer burden in Somalia.

### Quality Assessment

The methodological quality of the included studies was assessed using the JBI Critical Appraisal Checklist for Analytical Cross-Sectional Studies [17]. This validated tool consists of eight criteria that evaluate key aspects of the study design and execution: clarity of inclusion criteria, description of study subjects and setting, validity of exposure measurement, objectivity of condition measurement criteria, identification of confounding factors, strategies to address confounding factors, validity of outcome measurement, and appropriateness of statistical analysis. Two independent reviewers (MMH and MMA) conducted the quality assessment, and disagreements were resolved through discussion or consultation with a third reviewer (NID).

## Results

### Study Overview

**Fig. 2:**
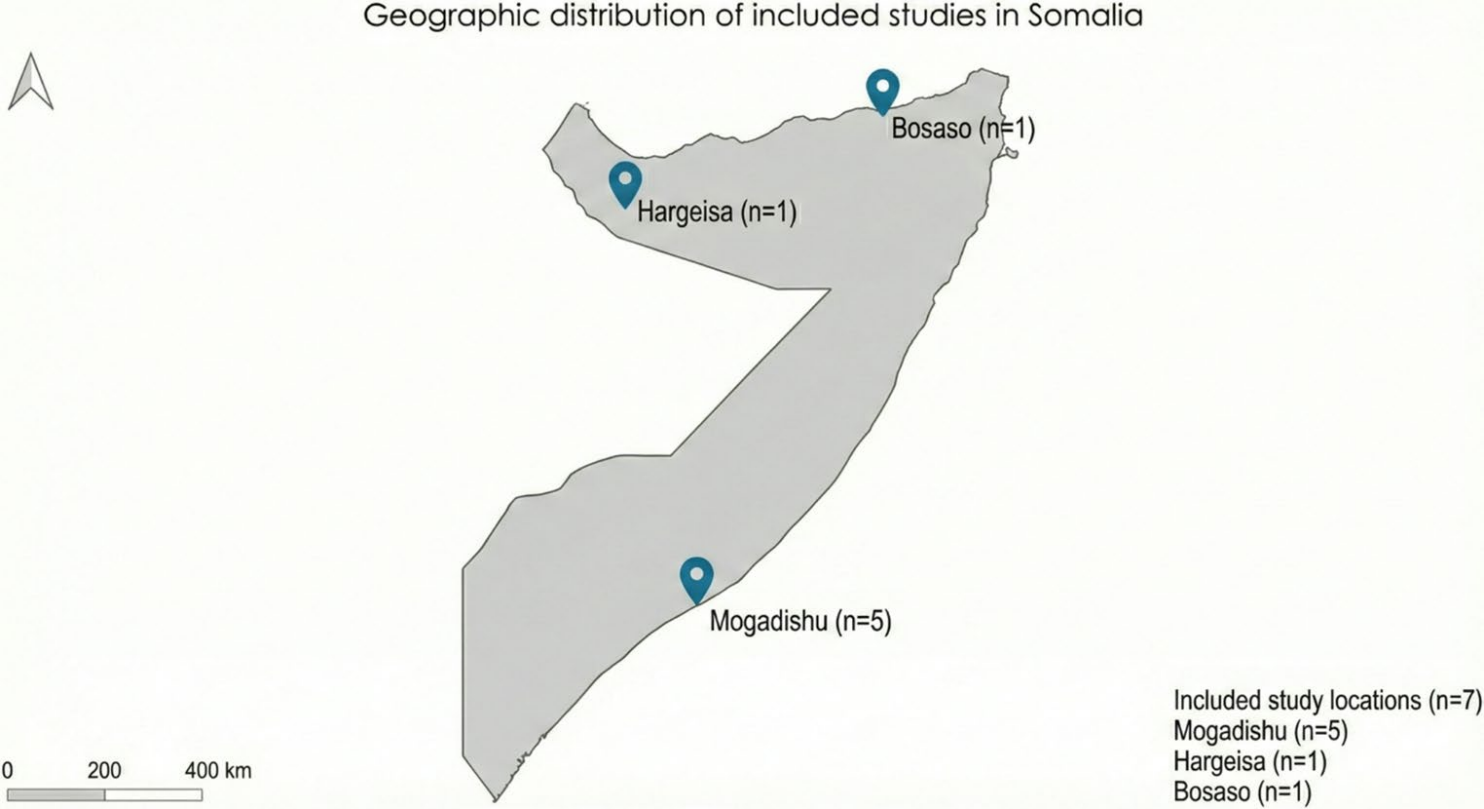
Geographic Distribution of Included Studies in Somalia

This systematic review included seven cross-sectional studies published between 2019 and 2024, all of which were conducted in Somalia (Table 1). The studies were geographically distributed across three regions (Fig. 2). Mogadishu (*n* = 5), Hargeisa (*n* = 1), and Bosaso (*n* = 1). The total sample size across all studies was 3,797 participants, with individual study samples ranging from 133 to 1,306. The studies targeted diverse populations, including cancer patients, healthcare professionals, and women of reproductive age [12, 13, 18–22]. The studies addressed various aspects of cervical cancer in Somalia, with research objectives broadly categorized into three domains: epidemiological assessment of cervical cancer prevalence, evaluation of knowledge, attitudes, and practices among healthcare professionals, and assessment of knowledge, attitudes, and practices among women. The temporal distribution of these studies enables the examination of potential changes in cervical cancer awareness, prevalence, and healthcare approaches over five years in Somalia [19, 21, 22].

**Table 1:** Barriers and attitudes toward cervical cancer screening in Somalia

Author(s)	Year	Study objective	Study design	Sample size	Population/setting	Region	Key findings	Recommendations
Nour & Alemayehu	2024	To assess knowledge, attitude, and associated factors of cervical cancer among reproductive-aged women	Cross-sectional study	270	Reproductive-aged women residing in Hargeisa	Hargeisa, Somalia	43.7% had good knowledge of cervical cancer risk factors/symptoms; 46.3% had a positive attitude	Increase awareness and education; improve access to screening and prevention
Altunkurek et al.	2022	To assess knowledge and attitudes of healthcare professionals toward early diagnosis of cervical cancer	Cross-sectional study	280	Healthcare professionals in a training and research hospital	Mogadishu, Somalia	Only 22.1% received cervical cancer training; only 2% of female healthcare professionals had Pap smear; HPV knowledge had highest score (69.6%)	Train healthcare professionals (especially female providers); address barriers through awareness + accessible services
Walz et al.	2022	To assess healthcare workers’ knowledge/attitudes/practices on breast & cervical cancer screening and identify barriers	Cross-sectional study	469	Healthcare professionals & students from hospitals	Mogadishu, Somalia	73% had good cervical cancer knowledge; 89% disagreed HPV vaccine was available; 30% had conducted screening	Expand HPV vaccination access; improve screening infrastructure and availability
Abdallah	2019	To assess knowledge, attitudes, and practices on cervical cancer prevention among adult females	Cross-sectional study	133	Women of reproductive age attending healthcare services	Bosaso, Bari, Somalia	97.7% had heard of cervical cancer; 88.4% knew women should be screened; misconceptions existed (e.g., transmissibility beliefs)	Strengthen awareness programs focusing on symptoms, transmission misconceptions, risk factors

### Prevalence of Cervical Cancer and HPV in Somalia

The burden of cervical cancer in Somalia was quantified in three studies that reported prevalence data [18, 20, 22]. Cervical cancer constituted 6.7% of all cancers and 13.3% of cancers among women, based on an analysis of 1,306 cancer patients diagnosed at STRTEH hospital in Mogadishu between 2017 and 2019 [18]. A more recent and focused study conducted in Mogadishu between December 2020 and February 2021 reported a substantially higher prevalence of 15.7% among 1,150 women aged 25–50 years who underwent screening [20]. This study used multiple screening methods, revealing varying positivity rates: 7.6% for Visual Inspection with Acetic Acid (VIA) and 5.1% for Pap smear, with a statistically significant difference between these methods (*p* = 0.004). Notably, 18.4% (27/146) of women had positive results on both VIA and Pap smear tests, while 73.9% (108/146) of VIA-positive women were confirmed to have intraepithelial cervical neoplasia (CIN) upon colposcopy and biopsy [20]. Critical insights into the prevalence of HPV, the primary etiological agent of cervical cancer, revealed that among 189 cervical samples collected from women of reproductive age in Mogadishu, 31.7% tested positive for HPV DNA [22]. More concerning was the finding that 19.6% of the samples contained high-risk HPV (hrHPV) types, with HPV16 (13.8%) and HPV18 (5.3%) being the most prevalent oncogenic strains [22].

### Knowledge, Attitudes, and Practices among Healthcare Professionals

Two studies specifically examined healthcare professionals’ knowledge, attitudes, and practices regarding cervical cancer in Mogadishu [12, 13]. Among 469 healthcare professionals comprising medical doctors, nurses, midwives, radiologists as well as health science students, 73% demonstrated good knowledge of cervical cancer [13]. Attitudes toward the clinical importance of the disease were overwhelmingly positive, with over 98% of respondents agreeing that cervical cancer is a “very serious” disease and that screening is an “essential part” of women’s healthcare [13]. However, significant attitudinal barriers were identified regarding clinical prioritization: 92.9% of providers believed their patients had “more pressing health problems” than cancer screening [13]. Additionally, many professionals held the belief that screening was “too expensive” (68.4%) or that the procedures were “too difficult” (52.2%) to implement in their current environment [13]. Despite these positive general attitudes, practical experience was limited, with only 24% of respondents reporting having treated a cervical cancer patient and 30% having conducted screening for either breast or cervical cancer [13].

A prominent finding was that 89% of healthcare professionals disagreed that HPV vaccines were available to their patients [13]. Additional insights from a survey of 280 healthcare professionals (doctors, nurses, midwives, and health officers) working in a training and research hospital in Mogadishu revealed that only 22.1% of healthcare professionals had received specific training on cervical cancer during their vocational education [12]. Knowledge about HPV was relatively better, with a 69.6% success rate on HPV-related knowledge questions [12]. Yet proactive clinical behaviors remained low; only 29.6% reported providing education to their patients and 40% recommended Pap smear testing [12]. Perhaps most concerning was the finding that only 2% of female healthcare professionals had ever undergone a Pap smear test themselves [12]. Among those never screened, the primary behavioral deterrent was the belief that they did not need a test because they had “no complaints” (45.8%) [12].

### Knowledge, attitudes, and Practices among Women

Two studies assessed knowledge, attitudes, and practices regarding cervical cancer among Somali women in different regions [19, 21]. Among 133 women of reproductive age attending healthcare services in Bosaso, high general awareness was found, with 97.7% of women reporting having heard of cervical cancer and 88.4% understanding that women should be screened for it [21]. However, the statement regarding misconceptions about transmission requires nuance: while infection with Human Papillomavirus (HPV) is typically transmitted during sexual intercourse and is the primary risk factor for the disease, the misconception identified in the sources is the belief that the cancer itself is a contagious condition. In the Bosaso study, 40.9% of respondents incorrectly reported that cervical cancer (the malignancy, rather than the virus) is transmissible, believing it could be spread not only through sexual intercourse but also through casual contact with the sick [21]. More concerning findings regarding women’s knowledge in Hargeisa revealed that among 270 reproductive-aged women, only 43.7% (118 women) demonstrated good knowledge of cervical cancer risk factors and symptoms [19]. Attitudes toward the disease, encompassing underlying perceptions, beliefs, and predispositions, were mixed, with 46.3% expressing a positive attitude [19]. 57.8% of women agreed that early detection is good for treatment outcomes, and 55.2% believed it is possible to detect the cancer through screening before symptoms appear [19].

### Screening Methods and Practices

Only two of the seven studies (Table 2) reported specific screening methods for cervical cancer detection in Somalia [20, 22]. A comprehensive approach using multiple screening modalities was used: Visual Inspection with Acetic Acid (VIA), Pap smear, and colposcopy with biopsy for confirmation of positive results [20]. This study found varying positivity rates between screening methods, with VIA detecting more potential cases (7.6%) than Pap smears (5.1%), representing a statistically significant difference (*p* = 0.004). In this cohort of 1,150 women, all participants received both tests; VIA identified lesions in approximately 87 women, while Pap smears were positive in 59. These results highlight the performance of VIA, which the sources describe as a simple, inexpensive test with moderate sensitivity and specificity that provides immediate results [19]. The confirmation of 73.9% of VIA-positive cases through colposcopy and biopsy suggests that, while potentially yielding more false positives than Pap smears, VIA remains a valuable screening tool in resource-limited settings such as Somalia. HPV DNA testing using real-time quantitative polymerase chain reaction (RT-PCR) assays was used to detect HPV prevalence among women of reproductive age in Mogadishu [22]. This molecular approach represents a more advanced screening technology that can identify women at risk before any cellular changes occur. The finding that 31.7% of samples tested positive for HPV DNA, with 19.6% positive for high-risk HPV types, demonstrates the potential value of HPV testing in identifying at-risk populations and the urgent need for a national HPV vaccination program, which is currently non-existent in Somalia [22].

**Table 2:** Cervical cancer and HPV prevalence and screening outcomes in Somalia

Author(s)	Year	Study objective	Study design	Sample size	Population/setting	Region	Key findings	Screening methods	Recommendations
Garba et al.	2024	To determine HPV prevalence using DNA-based testing among reproductive-aged women	Cross-sectional study	189	Cervical samples collected and analyzed	Mogadishu, Somalia	HPV DNA prevalence 31.7%; high-risk HPV 19.6%; HPV16 13.8%; HPV18 5.3%	HPV DNA testing (RT-PCR)	Implement nationwide HPV screening & vaccination, especially rural areas; improve affordability/access
Siad	2023	To assess prevalence of cervical cancer and screening outcomes	Cross-sectional study	1,150	Women screened in Mogadishu (Dec 2020–Feb 2021)	Mogadishu, Somalia	15.7% tested positive for cervical cancer; VIA 7.6%, Pap smear 5.1%; biopsy-confirmed CIN common among VIA+	VIA, Pap smear, colposcopy & biopsy confirmation	Promote education on susceptibility; provide free screening and HPV vaccines
Tahtabasi et al.	2020	To determine type, frequency, and distribution of cancers in Somalia	Cross-sectional study	1,306	Cancer patients diagnosed at STRTEH hospital (2017–2019)	Mogadishu, Somalia	Cervical cancer 6.7% of all cancers; 13.3% of female cancers were cervical	–	Establish a national cancer registry system

### Barriers and Recommendations for Cervical Cancer Control

All seven studies provided recommendations for improving cervical cancer control in Somalia, with several common themes [12, 13, 18–22]. Infrastructure development has been emphasized in multiple studies, with specific recommendations for establishing a national cancer registry system to better monitor and respond to the cancer burden [18]. The need for accessible and affordable cancer treatment facilities throughout Somalia has also been advocated [22]. Expanding access to preventive services is another prominent recommendation. The need to improve access to HPV vaccination, particularly in rural areas, has been emphasized [13, 22]. Providing free screening services and HPV vaccines to enhance prevention efforts has been specifically recommended [20]. Education and awareness were consistently identified as critical needs across the included studies. Increasing cervical cancer awareness and education programs have been recommended [19], and suggestions have been made that these educational efforts should specifically emphasize signs and symptoms, transmission methods, and risk factors [21]. Healthcare professional education was a focus, with recommendations for training all healthcare professionals, with special attention to female providers who may serve as role models for cervical cancer screening [12]. In addition to these recommendations, the included studies identified several barriers to effective cervical cancer control. The limited availability of HPV vaccines and inadequate screening infrastructure have been highlighted [12, 13]. Low screening uptake related to sociocultural factors, cost, and limited-service accessibility was also noted, indicating barriers to screening acceptance in facility-based settings [12].

## Discussion

This systematic review identified seven cross-sectional studies examining cervical cancer in Somalia, revealing several significant findings with important implications for public health and policy. The primary findings include a substantial burden of cervical cancer among Somali women, a high prevalence of HPV infection with significant representation of high-risk types, limited knowledge and screening practices among both healthcare professionals and women, and various barriers to effective cervical cancer control. The prevalence of cervical cancer among Somali women ranges from 6.7% to 15.7%, with cervical cancer constituting 13.3% of all cancers among women. In facility-based studies, cervical cancer accounted for 6.7% of all cancers and 13.3% of female cancers, and screening positivity reached up to 15.7% among women attending screening services in Mogadishu; however, these figures derive from hospital- and clinic-based populations and should not be interpreted as population-level prevalence estimates for Somalia [18, 20]. This burden is concerning and likely reflects limited access to preventive services, including HPV vaccination and screening [23, 24]. The variation in prevalence estimates across studies may be attributed to differences in the study populations, methodologies, and geographic locations.

The high prevalence of HPV infection (31.7%) with a significant proportion of high-risk types (19.6%) is particularly concerning. The predominance of HPV16 (13.8%) and HPV18 (5.3%) is consistent with global patterns but represents a significant risk factor for cervical cancer in Somali women, while a clinic-based study found 31.7% of women tested were HPV-positive (with HPV16 and 18 most frequent), this high rate is from a limited sample and may reflect sampling bias. Nonetheless, the presence of oncogenic HPV types in that subset underscores the potential risk in Somalia if screening coverage remains low [22, 25, 26]. The high HPV prevalence in Somalia likely reflects limited access to HPV vaccination, inadequate screening programs, and various socioeconomic and cultural factors that may influence sexual health behaviors and health care-seeking patterns [27, 28]. Knowledge gaps among healthcare professionals represent critical barriers to effective cervical cancer control. Although 73% of healthcare workers demonstrated good theoretical knowledge of cervical cancer, practical experience was limited, with only 24% reporting having treated cervical cancer patients and 30% having conducted screenings [13].

This proportion of active practice is unexpectedly high given that the region faces a complete lack of functioning mammography equipment and a widespread unavailability of standard cervical cancer screening tools [13]. Such a disparity suggests that reported screenings likely rely on alternative diagnostic workarounds, including clinical histories or prohibitively expensive ultrasounds and biopsies, necessitated by a healthcare infrastructure severely damaged by decades of conflict [13]. Furthermore, while clinical attitudes are positive, over 96% of providers report lacking the essential technological or fiscal resources required to provide standard-of-care screenings [13]. The finding that only 22.1% of healthcare professionals received specific training on cervical cancer during their vocational education highlights significant gaps in medical education and professional development [12]. One limitation of the referred study is their inability to distinguish training received between doctors and other healthcare professionals including nurses and midwives. This is important because doctors typically are known to have deeper clinical knowledge about health events including cervical cancer diagnosis and treatment, whereas nurses often focus on preventive care, patient education, and supportive roles [29]. Notwithstanding, these gaps are particularly concerning, given the crucial role healthcare professionals play in cervical cancer prevention, early detection, and treatment [30, 31].

The low rate of cervical cancer screening among female healthcare professionals (only 2% had undergone Pap smear testing) is particularly troubling and suggests that barriers to screening affect even those who should be most informed about its importance [12]. Although, the study didn’t distinguish between doctors and other healthcare professionals in assessing the pap smear screening, the fact that only 2% out of the total that include 89 (31.8%) doctors had undergone screening is a very big concern that may reflect a huge educational failure. While cervical cancer screening is crucial, some healthcare professionals, including doctors, may not consistently perform or recommend it, citing factors like time constraints, perceived complexity, and the belief that gynecologists are better suited for the task [32].

This finding may reflect cultural and educational barriers, limited access to screening services, or competing healthcare priorities in resource-constrained settings [33]. Addressing these barriers among healthcare professionals is essential, as they serve as role models and advocates of preventive health behaviors in their communities. Knowledge of and attitudes toward cervical cancer among Somali women vary considerably across studies and regions. While general awareness was high in some regions (97.7% in Bosaso), detailed knowledge of risk factors and symptoms was limited (43.7% in Hargeisa) [19, 21]. The low knowledge level in Hargeisa may be since Hargeisa, while being an urban settlement, is smaller, less densely populated, and maintains stronger rural linkages, with nearby areas heavily involved in pastoralism and agriculture.

Another important factor is that 57.7% of the participants studied in Hargeisa has only high school, primary and Islamic education which have all been reported to be associated with poor knowledge [34, 35]. Misconceptions about disease transmission are common, with 40.9% of women in one study believing that cervical cancer is transmissible through sexual intercourse or contact with an infected person [21]. These knowledge gaps and misconceptions may contribute to stigma, fear, and reluctance to seek screening and treatment for the disease [36]. The limited implementation of screening programs is another significant barrier to cervical cancer control in Somalia. Only two studies reported specific screening methods, reflecting the nascent state of cervical cancer screening programs [20, 22]. Beyond screening availability, continuity of care for women who screen positive remains a critical but significantly neglected component of cervical cancer control in Somalia. In a study of 374 healthcare professionals and students in Mogadishu, 61.2% of respondents reported a specific lack of capacity to conduct patient follow-up after an initial screening [13].

This gap in the clinical continuum is exacerbated by a healthcare infrastructure severely damaged by decades of civil war, resulting in limited electricity coverage and the widespread absence of organized referral systems [13]. Consequently, many existing screening activities are opportunistic or isolated, occurring without a proper referral pathway to accessible and affordable treatment facilities [13]. The use of VIA, Pap smear, and HPV DNA testing represents a range of screening approaches that can be adapted to various resource settings. However, the limited reporting on screening methods suggests that systematic screening has not been widely implemented.

Emerging technologies may offer future opportunities to strengthen cervical cancer control in resource-limited settings such as Somalia. AI-assisted diagnostic tools and molecular HPV testing (such as real-time PCR) could help reduce dependence on specialized personnel and laboratory infrastructure [12]. Digital health and telemedicine platforms may also support remote consultation and follow-up care, addressing documented service disruptions [31]. While not yet widely implemented, these approaches represent promising directions for improving screening and continuity of care [13]. This systematic review had several limitations. First, all the included studies were cross-sectional, which limited our ability to assess temporal trends or causal relationships. Second, the studies were conducted in only three regions of Somalia (Mogadishu, Hargeisa, and Bosaso), potentially limiting the generalizability of the findings to other regions, particularly rural areas. Third, the heterogeneity in study populations, methodologies, and outcome measures limit our ability to conduct a quantitative synthesis or meta-analysis. Fourth, the relatively small number of included studies (*n* = 7) reflects the limited research on cervical cancer in Somalia, highlighting the need for more comprehensive and rigorous research.

Additionally, the possibility of publication bias cannot be excluded, as studies with null or negative findings may be underrepresented in the available literature. Based on these findings, several recommendations can be made to strengthen the cervical cancer control efforts in Somalia. First, establishing a national cancer registry system would enable better monitoring and response to the cancer burden [18]. Second, expanding access to HPV vaccination, particularly in rural areas, is essential for primary prevention [13, 22]. Third, improving screening infrastructure and providing free screening services will enhance early detection [20]. Fourth, comprehensive education and awareness programs targeting both healthcare professionals and the public are needed to address knowledge gaps and misconceptions [19, 21]. Fifth, training healthcare professionals, especially female providers, in cervical cancer screening and management would strengthen the healthcare workforce capacity [12]. Finally, addressing barriers to screening acceptance through culturally appropriate interventions is essential to improve screening uptake.

## Conclusion

This systematic review reveals substantial challenges in cervical cancer control in Somalia, based on evidence from facility-based studies and screening populations. Available data indicate a high burden of cervical cancer within hospital settings and a considerable presence of HPV infection among tested women, underscoring the ongoing public health concern. Our findings highlight significant gaps in healthcare infrastructure, professional training, and public awareness that contribute to cervical cancer remaining the second most frequent cancer among Somali women. Extremely limited screening coverage estimated at 1–2% of eligible women, combined with knowledge deficits among both healthcare providers and women, poses major barriers to effective prevention and early detection. Cultural misconceptions regarding HPV transmission further complicate screening acceptance. Based on these findings, we recommend establishing a national cancer registry, expanding HPV vaccination access, strengthening screening infrastructure, implementing comprehensive education programs, enhancing healthcare professional training, and addressing cultural barriers through targeted interventions. Collectively, these measures are essential to improving cervical cancer outcomes in Somalia’s resource-constrained healthcare system and reducing mortality from this largely preventable disease.

## Supplementary Information

Below is the link to the electronic supplementary material.

Supplementary Material 1 (DOCX 269 KB)

## Data Availability

Data sharing is not applicable to this article as no datasets were generated or analysed during the current study.
